# A Preliminary Exploration of the Placental Position Influence on Uterine Electromyography Using Fractional Modelling

**DOI:** 10.3390/s22051704

**Published:** 2022-02-22

**Authors:** Müfit Şan, Arnaldo Batista, Sara Russo, Filipa Esgalhado, Catarina R. Palma dos Reis, Fátima Serrano, Manuel Ortigueira

**Affiliations:** 1 Department of Mathematics, Çankırı Karatekin University, Çankırı 18100, Turkey; mufitsan@karatekin.edu.tr; 2 NOVA School of Science and Technology, NOVA University Lisbon, 2829-516 Caparica, Portugal; s.russo@campus.fct.unl.pt (S.R.); feo.cardoso@campus.fct.unl.pt (F.E.); mdo@fct.unl.pt (M.O.); 3 UNINOVA-CTS, NOVA School of Science and Technology, NOVA University Lisbon, 2829-516 Lisbon, Portugal; 4 NMT S.A., Parque Tecnológico de Cantanhede, Núcleo 04, Lote 3, 3060-197 Lisbon, Portugal; 5 Maternidade Alfredo da Costa, Rua Viriato 1, 1050-170 Lisbon, Portugal; palmareisc@gmail.com (C.R.P.d.R.); fatima_serrano@hotmail.com (F.S.); 6 Faculty of Medical Sciences, Nova Medical School, NOVA University Lisbon, 1169-056 Lisbon, Portugal

**Keywords:** uterine electromyography, fractional calculus, transfer function modelling, Cole-impedance model

## Abstract

The uterine electromyogram, also called electrohysterogram (EHG), is the electrical signal generated by uterine contractile activity. The EHG has been considered an expanding technique for pregnancy monitoring and preterm risk evaluation. Data were collected on the abdominal surface. It has been speculated the effect of the placenta location on the characteristics of the EHG. In this work, a preliminary exploration method is proposed using the average spectra of Alvarez waves contractions of subjects with anterior and non-anterior placental position as a basis for the triple-dispersion Cole model that provides a best fit for these two cases. This leads to the uterine impedance estimation for these two study cases. Non-linear least square fitting (NLSF) was applied for this modelling process, which produces electric circuit fractional models’ representations. A triple-dispersion Cole-impedance model was used to obtain the uterine impedance curve in a frequency band between 0.1 and 1 Hz. A proposal for the interpretation relating the model parameters and the placental influence on the myometrial contractile action is provided. This is the first report regarding in silico estimation of the uterine impedance for cases involving anterior or non-anterior placental positions.

## 1. Introduction

The EHG has been used as a modern tool for pregnancy monitoring and preterm risk evaluation [1,2]. It has been pointed out as having the potential to replace the tocogram in pregnancy monitoring given its superior sensitivity [3,4,5]. One of the advantages of the EHG technology is its non-invasive nature since the electrodes are in designated places in the abdominal surface. EHG electrodes capture the electric potential due to the uterine contractile activity, which is present throughout pregnancy and can be detected as early as 19 weeks of gestation [6]. Subjects with a high body mass index (BMI) reportedly may have poor tocography recordings due to the fat layer attenuation effect [7]. The EHG has been deemed to be less affected by the high BMI attenuation effect [5,8]. The question arose as to whether an anterior placenta could introduce signal EHG variations given the acquisition electrodes being in the projection line containing the involved myometrial tissue and the anterior placenta. Dermott et al. (2010) [9] reported no significant contraction differences in amplitude, frequency, and integral activity between anterior and posterior placental groups during labour. Contrarily, Kavšek el al. (1999) [10,11] observed a significant increase in the EHG contractions root mean square (RMS) value for subjects with non-anterior placenta (46.50 to 82.93 µV) and a noticeable decrease in the mean frequency (0.25 to 0.20 Hz) for the same group. The local hormonal inhibitory influence of the placenta in the surrounding uterine muscles is reported as the cause for the RMS reduction for anterior placental implantation. This research associated higher RMS results with lower mean frequency values. Basal uterine activity is, according to the work of Kavšek et al. (1999) [10], associated with increased mean frequency values, being the anterior uterine wall the main contributor for the EHG. On the other hand, Grgic et al. (2006) [12] reported no statistical significant differences between the two involved study groups for subjects in the mid- trimester of pregnancy using a bipolar EHG electrode arrangement. The used parameters were the median frequency and median amplitude of the EHG. The apparent discrepancy between these authors’ results may be related to conflicting electrical resistance calculations, namely the way the resistance is estimated, specifically if it is obtained from the real part or, otherwise, the absolute value of the impedance [13]. In an in vivo study, Gandhi et al. (2006) [14] reported an increase in the cervix electrical resistivity in pregnant women compared to non-pregnant subjects along with a trend where the resistivity increases with gestational age, suggesting that increased collagen content in the cervix weights over cervical hydration. The frequency band in this study extended from 4 kHz to 819 KHz. Roughly, given electrode areas and current path length, the electrical resistance in the second trimester was reported as being around 4 KΩ compared to 6 KΩ in the third trimester. Somewhat contradictory results were obtained in another study of Avis et al. (1996) [13], where a small sample of cervix tissue was used to evaluate the electric resistance. The results of this pilot study showed a lower R/S ratio and RC time constant for the term group compared with the preterm group. In this study, R represents the resistance of the extracellular space, S the resistance of the intracellular space, and C the capacitance of the tissue’s cells membranes for the circuit model under study.

Fractional calculus is a powerful tool for modelling phenomena in science and engineering. Although its history dates back as far as integer-order calculus, its progress over the last thirty years has been enormous and has become the object of great interest of not only of mathematicians but also other researchers in science and technology [15,16,17,18,19,20,21,22]. Fractional-order calculus has been used to generalize various types of controllers, including internal model controllers [23]. Biochemical processes modelling has been implemented using fractional calculus to facilitate the interpretation of its complex mechanisms that often are not intuitive to understand [24]. Fractional calculus can model numerous real-world phenomena better than integer-order calculus. The fractional-order models, for example, present better capabilities for electrochemical capacitors characteristics approximation than models based on the integer order [16,25,26,27,28]. Fractional-order circuit models and systems are emerging fields that have resulted of importing concepts from fractional calculus into electrical circuit theory [29]. The single-dispersion Cole-impedance model was introduced by Cole in 1942. Despite its fractional character, fractional calculus was not taken into consideration when it was proposed [30]. This model is composed of three circuit elements: a high-frequency resistor $R_{0}$, a resistor $R_{1}$, and a constant phase element (CPE), or fractional capacitor, with capacitance $C_{1}$ and positive real order $\alpha_{1}$ leading to its fractional character. This model is simple and a good fit for numerous data sets collected from biological tissues and materials. By combining Cole circuit elements, the double- and triple-dispersion Cole models were created to improve fitting characteristics [28,31,32,33,34,35]. These Cole models have been applied for monitoring necrosis of human tumour xenografts during and/or after hyperthermia treatment [36]; investigating age-related changes of dentine to create non-destructive test methods [37]; assessing quality of red blood cell suspensions under storage [38]; modelling lung, beef, and veal meat impedance [39,40,41]; and numerous others [34].

On the other hand, for describing the fractional character of these fractional calculus-depending models, it is natural to consider the CPE described by the current-voltage relationship in the following differential equation:

(1) $$i\left(t\right)=C\frac{d^{\alpha }}{dt^{\alpha }}v\left(t\right),\;$$

where functions i(t) and v(t) are the time-dependent current and voltage, *C* is the capacitance, measured in *Farad*/$sec^{1-\alpha }$, and $\frac{d^{\alpha }}{dt^{\alpha }}$ represents the fractional derivative [42]. For describing the constant phase element in Equation (1), we use the Grünwald–Letkinov fractional derivative defined on the whole real line, which is given by [28]:

(2) DGLαv(t)=limh→0*∑k=0∞(−1)k(αk)v(t−kh)hα, t∈R, α∈R. 

**Figure 1:**
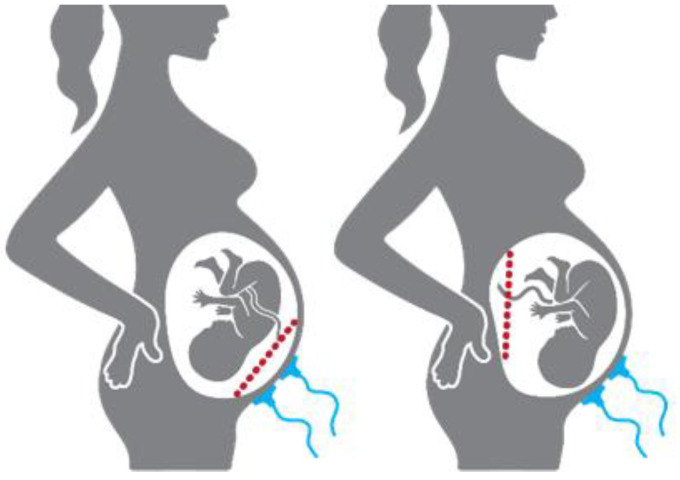
Placental position relatively to the acquisition electrodes (blue): (**Left**) anterior placenta (red dash line); (**Right**) an example of non-anterior placental position (red dash line).

In this study, the placental position influence on the EHG spectral parameters will be investigated using a preliminary exploratory methodology. The anterior placenta is positioned at the front wall of the uterus and is therefore in the projection of the myometrium and the acquisition electrodes. This placenta position has the potential to influence the EHG characteristics. The other placenta positions, herein referred to as non-anterior, are not in the electrode’s projection. Figure 1 provides a simplified illustration regarding placental position relatively to the acquisition electrodes. Tissue impedance has been widely used as a biomarker for biological tissues, such as animal tumour growth and status evaluation, or vegetal non-destructive fruit-ripening assessment [34,36,37,43,44].

The main goal is to obtain the electrical impedance fractional estimation in silico model of the uterine tissue using non-invasively obtained data. Considering that anterior placenta stands in the alignment of the electrodes and the myometrium, its effect on the impedance model is concurrently studied. A tentative interpretation of the results is provided. To the best of the authors’ knowledge, this is the first study using the mentioned methodology for the uterine impedance estimation modelling.

## 2. Materials and Methods

**Figure 2:**
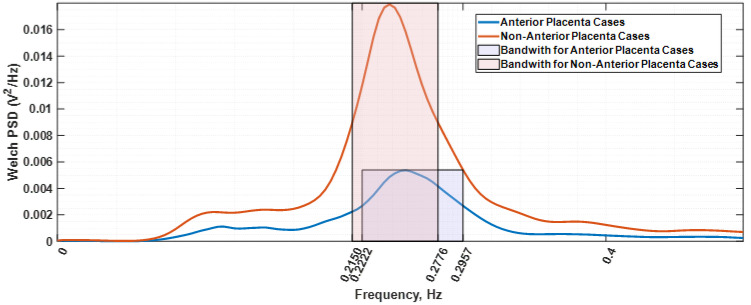
Welch periodogram estimations of the average spectra for the contraction’s subjects with anterior (blue colour) and non-anterior placenta (orange colour). The respective bandwidths are shown in the shadow rectangular areas.

The used data were provided by the Iceland Uterine Electromyography database [45], comprising 121 EHG recordings. Each record includes about 30 min of data from a 16-channel electrode configuration, located in the abdominal surface. Database parameters are described in Table 1. To reduce maternal respiration and common mode noise interference, a bipolar arrangement between channels 4 and 10 was used. The selection criteria for this electrode arrangement can be found in Esgalhado et al. (2020) [46]. A uterine contraction detector, previously developed under the Uterine Explorer project [46,47,48,49], [50] processed the mentioned database. The obtained contraction data pool was, subsequently, the input for an unsupervised clustering classifier. The obtained clusters were identified using a retrospective correlation study involving the contraction occurrence rate and amplitude. This led to the characterization of the Alvarez and Braxton-Hicks contraction types, with the former separated into Alvarez low (AlvL) and Alvarez high (AlvH) categories [46]. For the herein presented work, both the AlvL and AlvH contraction sets could have been selected. The former was chosen. The other possible option of using the extracted Braxton-Hicks (BH) contraction dataset was rejected given that, compared with the Alvarez waves, the BH spectral estimation was found to be less smooth and would incur in higher model-fitting estimation errors and overly complexity in the obtained circuit model. The other reason for the Alvarez waves selection was that they are generated in local myometrium areas, and thus, the tissue under study is a restricted zone. Subsequently, the Alvarez dataset was divided into two categories regarding the subject placental position: anterior and non-anterior placenta. Alvarez waves have been deemed as being labour triggers, while BH components are associated with normal pregnancy progress [51,52]. Only contractions for subjects between 36 and 41 gestational weeks were selected. This exclusion criterium was established to narrow the observation interval regarding uterine maturation. Anterior placenta is in the projection of the electrodes’ location and the myometrium, and the goal here is to explore its impact on the electrical impedance of the overall tissue as far as seen by the selected electrodes. Welch power spectral density [53] of all the selected AlvL contractions were obtained and divided in two groups depending on the placenta position and subsequently averaged. Figure 2 represents the resulting spectra, a Welch periodogram estimation, where it is shown that for the non-anterior placenta case, the frequency power spectral peak (0.2124 Hz) is reduced relatively to the anterior placenta case (0.2539 Hz). The bandwidth of the anterior placental case is 0.0735 Hz, whereas for the other case, it is 0.0626 Hz. It therefore becomes clear that the anterior placenta plays a filtering role on the EHG. Additionally, it is also observed in Figure 2 an attenuation effect for the anterior placenta case that translates in an amplitude decrease in the PSD estimation for this case. This effect has also been reported elsewhere [10].

**Figure 3:**
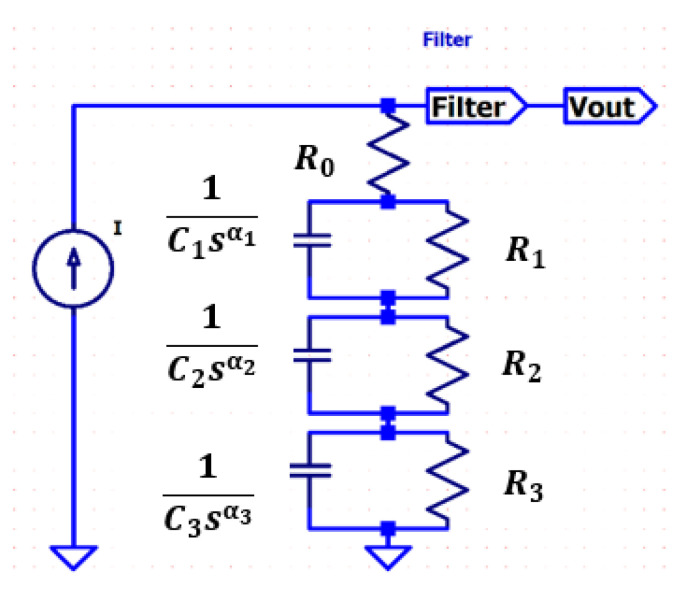
Proposed triple-dispersion Cole model. The filter, *F*, accounts for the used filter for processing the automatically detected contractions [48].

A fractional-order transfer function is obtained from Figure 3; a triple-dispersion Cole-impedance model providing a best fit to the data given by Figure 2 is to be implemented for the anterior and non-anterior placental position cases. This model produces an electric circuit representation that leads to the assessment of the placental implantation effect on the EHG. It is assumed that the current source produces a Dirac-like current waveform for which the Laplace transform has a unity value.

In this work, a nonlinear least square fitting (NLSF) procedure based on the trust-region-reflective approach was performed to obtain parameters of the model in Figure 3. The Cole order model selection was performed through a trial-and-error procedure, where the criteria was to obtain the lowest model order that would fit the data within an acceptable error level. Higher model orders would result in better fitting but at a computational cost beyond a reasonable cost–benefit relation. Additionally, any possible model interpretation within the electrophysiology framework will be easier for lower model orders. The novelty of this work resides in using the spectrum of the output voltage related to the uterine contraction electromyogram for the Cole parameters estimation. The used NLSF algorithm is sensitive to the initially selected constants and lower and upper bound values for the model extracting the parameters within an acceptable estimation error. The drawback of this new approach for impedance calculation is that the obtained values should be rescaled for realistic ones. However, the impedance plot waveshape is not modified by this rescaling operation. The stability of the system of the proposed model is evaluated as well as the total error between the estimation function and the spectral function.

Fractional circuits have been used in biomedicine and biology for modelling tissue impedance [34,44,54]. Typically, the Cole-impedance model is employed, possibly in multiple dispersion modules. The triple-dispersion Cole model shown in Figure 3 consists of four resistors $\left(R_{j}\left(j=0,\ldots ,3\right)\right)$ and three constant phase elements (CPEs) $\left(C_{k}\left(k=1,2,3\right)\right)$ as follows:

(3) $$H\left(s\right)=R_{0}+\frac{R_{1}}{1+R_{1}C_{1}s^{\alpha_{1}}}+\frac{R_{2}}{1+R_{2}C_{2}s^{\alpha_{2}}}+\frac{R_{3}}{1+R_{3}C_{3}s^{\alpha_{3}}}$$

where it is assumed that s=iω. Furthermore, it is supposed that all $R_{j}$, $C_{k}$, and $\alpha_{k}$ are the unknown real nonnegative parameters since there is no physical meaning for negative valued resistors, capacitors, and $\alpha_{k}$ Equation (4).

The parameters to be obtained in Equation (3) are real values providing best fit for anterior and non-anterior placental position cases. These parameters were extracted through an algorithm involving the MATLAB function *lsqnonlin* for the implementation of this NLSF process based on [55,56]. The *lsqnonlin* function is specified by the goal function as follows:

(4) $$f\left(x\right)=f\left(R_{j},C_{k},\alpha_{k}\right),\;\;\left(j=0,1,2,3;\;k=1,2,3\right)$$

expressed by:

(5) $$f\left(x\right)=\sum_{n=1}^{L}\left|G\left(x;iw_{n}\right)-y\left(w_{n}\right)\right|$$

where *x* is the vector of parameters ($R_{j},$ $C_{k},$ $\alpha_{k})$ to minimize f(x), y is the spectral data, L is the total number of the experimentally angular frequencies $\omega_{n}$ for each data point in *y*, and *G* is the estimation function:

(6) $$G\left(x;i\omega_{n}\right)=\left(H\left(x;i\omega_{n}\right)F\left(i\omega_{n}\right)\right)\left(\bar{H\left(x;i\omega_{n}\right)F\left(i\omega_{n}\right)}\right)$$

where *F* is the band pass filter transfer function given by Equation (7), with coefficients $b_{m}$ and $a_{m}$.

(7) $$F\left(s\right)=\frac{\sum_{m=0}^{17}b_{m}s^{17-m}}{\sum_{m=0}^{17}a_{m}s^{17-m}}$$

**Figure 4:**
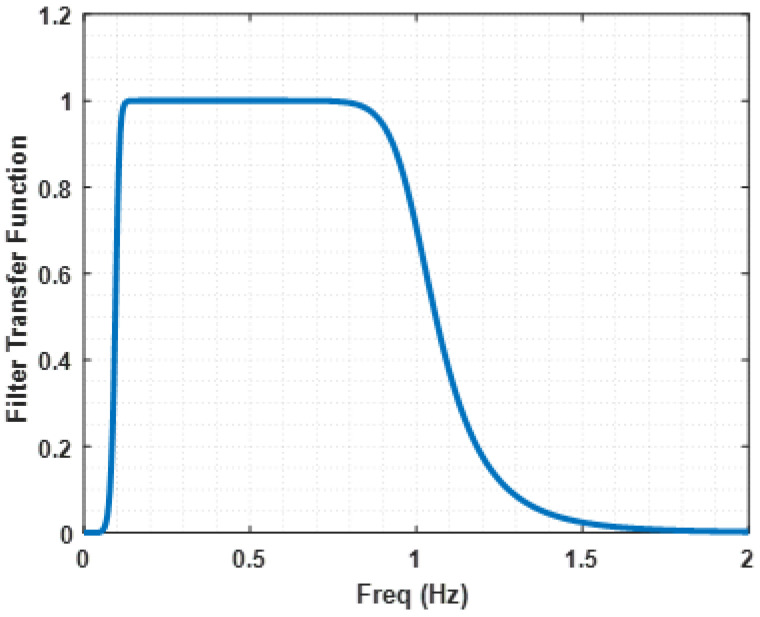
Filter transfer function, *F*. This filter represents the one used in the automatically detected contractions processing [48].

The *F* filter transfer function is represented in Figure 4. As mentioned in [48], this filter operates over the data to reduce interference.

Moreover, NLSF processing needs initial points, namely lower and upper bounds for each parameter in Equation (4). All lower bounds for $\alpha_{k}$ are selected to be equal to 1, and all the upper bounds are restricted to be 2. The lower and the upper bound values for $R_{j}$ are specified by 0.001 and 5000 values, respectively. The lower and the upper bound values for $C_{k}$ are 0.00001 and 50, respectively. The initial point for each parameter was determined within the ranges between its lower bound and upper bound in all cases.

The optimal values of parameters $\left(R_{j},C_{k},\alpha_{k}\right)$ of the model in Equation (3) are determined using the trust-region-reflective approach for performing the actual minimization of f(x). In addition to this, for each case of the anterior and the non-anterior placenta positions, the algorithm shows the location of the poles of model Equation (3), which yields the stability of the system. The total error between the experimental data and the estimate function *G* in Equation (6) is also obtained, which determines the good quality of the approximation of the estimate function G to the spectrum.

## 3. Results

**Figure 5:**
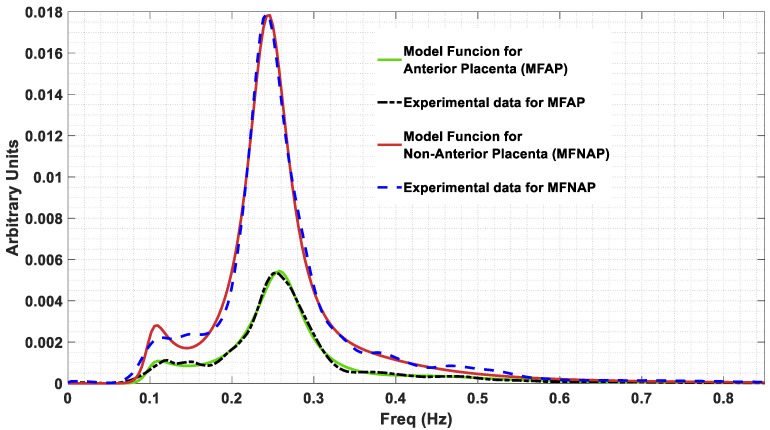
Model function for anterior placenta (MFAP, green colour) and the model function for non-anterior placenta (MFNAP, red colour). The experimental data spectra for MFAP (black colour) and the MFNAP (blue colour) are represented.

**Figure 6:**
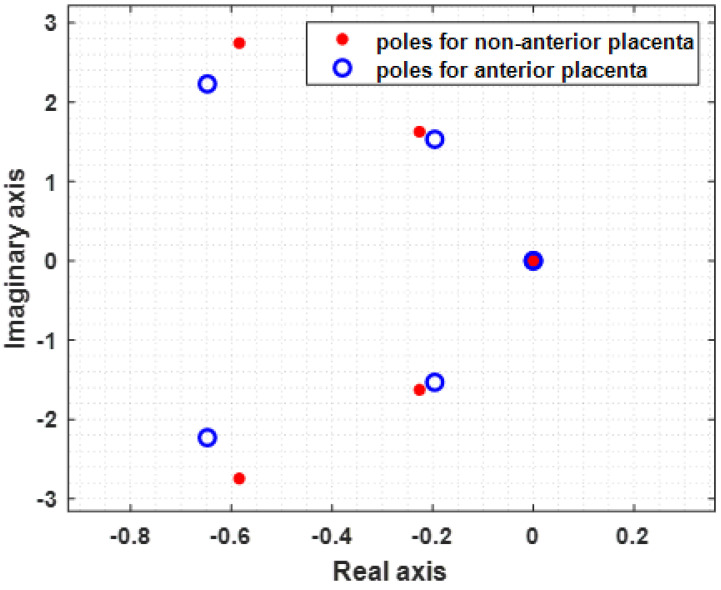
Poles of *H*(*s*) for the non-anterior placenta case (blue) and anterior placenta case (red), showing system stability. All poles are in the left half Argand plane.

As a result of the optimization fitting procedure applied to the model given by expression (3), the values in Table 2 were extracted. The obtained α values belong to the interval 1 < α < 2. The obtained values for the resistances and the capacitances are the ones the estimator produced and do not represent the physical values, for which a rescaling would be necessary, based on some known values for these two variables. The total error between the estimate function G defined by Equation (6) and the spectrum for the anterior placental position is 1.6456 $\times \;10^{-6}$, while for the non-anterior placental position, it is 1.4436 $\times \;10^{-5}.$ Figure 5 shows represented the model function for anterior placenta (MFAP, green colour) and the model function for non-anterior placenta (MFNAP, red colour). Additionally, the experimental data spectra for MFAP (black colour) and the MFNAP (blue colour) are represented. A good fitting was obtained. Since all *H*(*s*) poles are located on the left-half plane, as shown in Figure 6, the system with the proposed model is stable.

The time constants for the three Cole dispersion models are obtained by:

(8) τk=(RkCk)1αk; k=1, 2, 3 

Using the classic formulation, the cut-off frequency is obtained:

(9) ftauk=1(2πτk)

Table 3 represents the time constants and cut-off frequencies for each of the single-dispersion cell in the Cole model circuit of Figure 3. To note that *τ*_1_ values in both placenta position cases represent a long-range process component of more than half an hour, corresponding to very low cut-off frequencies.

**Figure 7:**
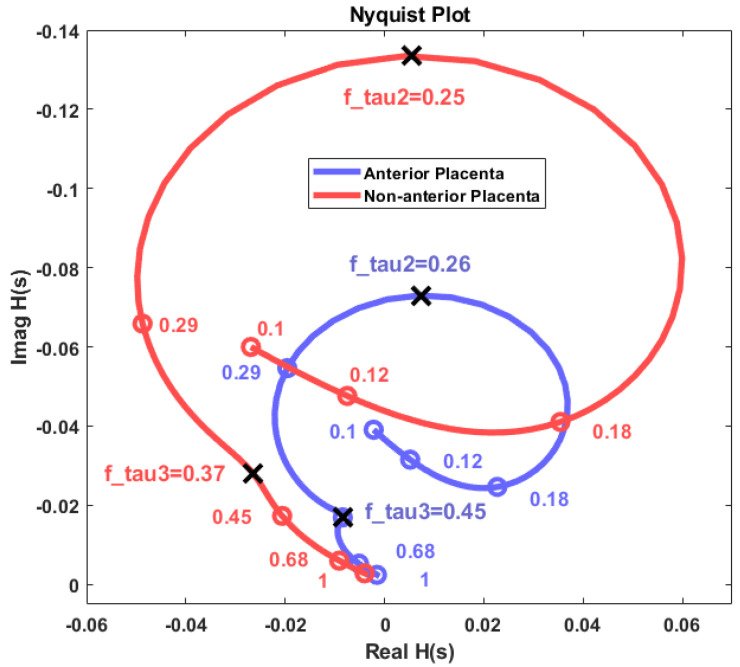
Nyquist diagram for the obtained impedance estimation for the placental and non-anterior placental cases. Axes are not scaled to physical resistance (real axis) and reactance (imaginary axis) values.

Figure 7 shows the Nyquist diagram-estimated impedance plot for the non-anterior placenta (red) and anterior placenta cases (blue). The curves represent frequency variation from 0.1 Hz to 1 Hz. The mark circles represent frequency points for the impedance estimations. Cut-off frequencies for both cases are represented and correspond to notches in the impedance curves. The long-range cut-off frequencies corresponding to *τ*_1_ have been excluded. The real and imaginary impedance axes do not represent the physical resistive or reactive components of the myometrial or myometrial plus placental tissue. The herein presented method is based on a spectral output, from which the obtained physical impedance values cannot be achieved. However, the impedance curve waveshape remains the same. Rescaling the impedance axes to physical values is possible if some physical impedance points are known. The 0.45-Hz cut-off frequency in the non-anterior placenta case is associated with a sharp impedance curve variation if compared with the corresponding 0.37-Hz cut-off frequency in the anterior placental case. This indicates that the time constant *τ*_2_ has more impact in the impedance variation for the anterior placental case.

**Figure 8:**
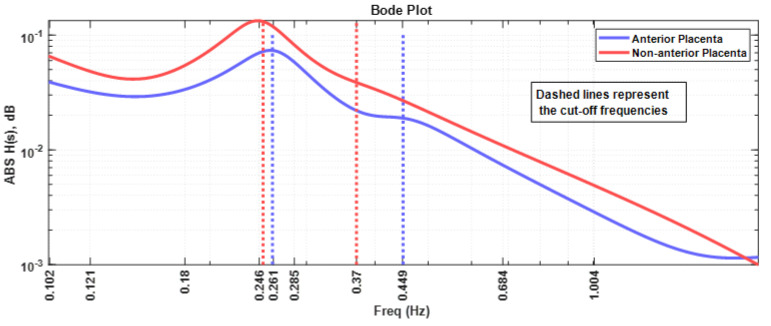
Bode plot of the myometrial impedance for the anterior placental and non-anterior placental cases. The cut-off frequencies are represented by the dash lines with the same colour as the respective curves.

Figure 8 represents the Bode plot of the absolute value of the impedance for both placenta position cases. All the comments presented for Figure 7 apply in this case. It should be noted that the anterior placental case impedance curve is roughly 10 dB below the non-anterior placental case.

## 4. Discussion and Conclusions

The obtained fractional circuit accurately represented the original spectral estimation for the anterior and non-anterior placental cases using only a triple-dispersion Cole model. This emphasizes fractional systems as an efficient tool to model complex electrophysiological phenomena, in this case, the uterine tissue impedance. It is also indicative of the potential fractional nature of the electrical characteristics of the uterine tissue. The presented study estimated, non-invasively, uterine tissue impedance using EHG data collected in the abdominal surface and projected to the spectral domain. The elegancy of the method has, however, the drawback of not being able to estimate the physical values of the tissue impedance. What is obtained is a Nyquist plot of the uterine impedance model. Nevertheless, a scaling to realistic impedance values is possible if actual resistance and reactance value pairs are known, via, for instance, in vitro or in vivo experiments.

During early human pregnancy, the trophoblast, an embryonic structure that will originate the placenta, invades the decidualized endometrium up to the inner third of the myometrium. It migrates in a retrograde direction along the spiral arteries, transforming them into large-diameter conduit vessels of low resistance [57]. Uteroplacental vessel development has been reported to occur in two phases: the first represents the decidual segments of spiral arteries at 8 to 10 weeks of gestation, invading the border between the decidua and myometrium [58]. The second stage, at 16 to 18 weeks, involves some intramyometrial spiral arteries invasion [58]. In a healthy pregnancy, this physiological transformation or remodelling is characterized by a gradual loss of the normal musculoelastic structure of the arterial wall that converts narrow-lumen, muscular spiral arteries into dilated, low-resistance uteroplacental vessels [57,59,60,61,62].

Our results show different myometrium frequency dependent impedance curves for the anterior placenta compared to non-anterior placenta case. In view of these results, it would be possible to hypothesize that:

- The mentioned remodelling could be in favour of a decreased myometrial impedance in cases where the placenta is anterior as a result of large uteroplacental vessels implantation, allowing increased placental blood flow [63]. This is in accordance with the Nyquist plot results in Figure 7, whereas the impedance range variation for both the resistance and reactance for the anterior placental case is lower compared to the non-anterior placental case.
- Regarding the intriguing increase in the frequency for the peak impedance value (Figure 8) in the anterior placenta case (0.261 Hz), relative to the non-anterior placenta case (0.246 Hz), further studies are required in this respect. The herein presented fractional circuit model may be beneficial for the understanding of this behaviour.
- Lower energy levels for anterior placenta, as reported in Figure 2 and Figure 5, may be due to local hormonal inhibitory influence of the placenta that blocks the propagation of uterine contractile activity [10]. Kanda et al. [64] concluded that, in rats, the muscular activity in the placental region is significantly inhibited until the last stage of pregnancy.
- Low energy levels identified in our study may also be a result of blocked propagation of electrical activity from the cells of the non-placental region to the placental region [10] presumably to avoid placenta abruption.

Our results are in accordance with Kavšek et al. [10], who also verified lower RMS values for EHG contractile activity in the anterior implantation placental site. According to their study, anterior uterine wall is the main source of the EMG activity, and as a result, the contractions of the posterior uterine wall do not affect the detected signal significantly.

Contrary to the study of Kavšek et al. and the herein presented one, Dermott et al. [9] reported no significant differences in frequency or power density values between anterior and non-anterior placental cases. However, this last study was performed for women in labour, a different population from both the herein presented work and the study of Kavšek et al.: the third pregnancy trimester only, before delivery.

The field of vegetal and animal fractional tissue modelling has presented interesting outcomes. Likewise, fractional calculus has the potential to explore and model myometrium electrophysiological phenomena. This could be an important contribution in areas such as pregnancy monitoring, preterm risk evaluation, and uterine electrophysiological research.

## Figures and Tables

**Table 1:** The Iceland Database Parameters.

*Parameters*	*Mean* ± *Standard Deviation*
*Maternal age (years)*	28.87 ± 5.63
*Gestational age at delivery (weeks)*	39.76 ± 1.40
*Pre-gestational BMI* $\left(\mathrm{Kg}\cdot m^{-2}\right)$	25.82 ± 4.80
*Gravidity*	2.62 ± 1.45
*Parity*	0.91 ± 0.92
*RMS (mV)*	0.04 ± 0.02
*Minimum RMS (mV)*	0.02
*Maximum RMS (mV)*	0.13
*Anterior Placenta*	24 subjects (70 recordings)
*Non-Anterior Placenta*	21 subjects (51 recordings)

**Table 2:** Component estimated values for *H*(*s*).

Case	R_0_ (Ω)	R_1_ (Ω)	R_2_ (Ω)	R_3_ (Ω)	C_1_ (*F*/ $\sec^{1-\alpha_{1}}$)	C_2_ (*F*/ $\sec^{1-\alpha_{2}}$)	C_3_ (*F*/ $\sec^{1-\alpha_{3}}$)	α_1_	α_2_	α_3_
Anterior Placenta	1.00 × 10^−3^	5.00 × 103	27.30 × 10^−3^	5.10 × 10^−3^	22.61	16.38	46.37	1.548	1.850	1.694
Non-Anterior Placenta	1.80 × 10^−3^	4.93 × 10^3^	15.70 × 10^−3^	3.20 × 10^−3^	40.30	25.51	50.00	1.387	1.838	1.764

**Table 3:** Time constants and cut-off frequencies for *H*(*s*).

	Anterior Placenta	Non-Anterior Placenta
*τ*_1_ (s1α1)	6.60 × 10^3^	1.83 × 10^3^
*τ*_2_ (s1α2)	0.609	0.648
*τ*_3_ (s1α3)	0.356	0.431
Cut-off freq. for *τ*_1_ (Hz)	2.41 × 10^−5^	8.66 × 10^−5^
Cut-off freq. for *τ*_2_ (Hz)	0.26	0.25
Cut-off freq. for *τ*_3_ (Hz)	0.45	0.37

## Data Availability

The dataset used in this work is open access: https://physionet.org/content/ehgdb/1.0.0/ (Last accessed on 30 October 2021).
